# Global research trends in West Nile virus from 1943 to 2016: a bibliometric analysis

**DOI:** 10.1186/s12992-017-0284-y

**Published:** 2017-08-03

**Authors:** Samah W. Al-Jabi

**Affiliations:** Department of Clinical and Community Pharmacy, Faculty of Medicine and Health Sciences, An-Najah National University, Nablus, 44839 Palestine

**Keywords:** West Nile virus, WNV, Bibliometric, Scopus, Citations

## Abstract

**Background:**

West Nile virus (WNV) is an emerging infectious disease which is most commonly transmitted to humans through mosquito, and is considered a major public-health problem worldwide. The aim of the current study is to bibliometrically analyze the quantity and quality of publications indexed in Scopus from different countries to reveal the characteristics of global research output regarding WNV.

**Methods:**

This study is a bibliometric analysis based on the Scopus database. This study focused on identifying WNV publication trends with regard to publication year, publication type, prolific countries, language of publication, as well as, prolific journals, citations, and collaboration patterns.

**Results:**

A total of 4729 publications were considered in this study, which were published between 1943 and 2016. The annual quantity of literature published before 2000 followed a low rate of research growth; while the quantity of publications after 2000 were published in a stage of rapid development. The country with the greatest number of publications in WNV research field was the USA with 2304 (48.7%) publications, followed by France with 224 (4.7%) publications, and Canada with 222 (4.7%) publications. Contributions from low- and middle-income countries (LMIC) were considerably small, that is, (*n* = 519 publications; 11%). All publications related to WNV achieved *h*-index of 140 and were cited 124,222 times. The median [interquartile range] number of citations per article thus amounts to 9 [2-28]. The USA had the highest *h*-index of 131. *Emerging Infectious Diseases* is the most productive journal with 227 articles, followed by *Journal of Virology* with 162 publications. The result designated that *Centers for Disease Control and Prevention* was ranked the first in terms of publication output, followed by *National Center for Emerging and Zoonotic Infectious Diseases*.

**Conclusions:**

There is an obvious trend of WNV research after 2000, and countries with high income have more contributions in WNV research field. The research output is low among LMIC. The USA produced the largest number of publications. The *Centers for Disease Control and Prevention* obtained the leading position of the institutions in terms of publication output. In general, this study not only presents a full view of global WNV research, but also can contribute for future further research in this field.

## Background

West Nile virus (WNV) is a mosquito-borne infection that is transmitted to humans by mosquito [1], and it is considered as a causative agent of the illness that represents a major public-health problem worldwide [2–4]. In 1937, WNV was first isolated from a patient’s blood in the West Nile region of Uganda [5]. Then, the virus is extensively distributed in Mediterranean region, Africa, Asia, and east Europe [6–8], and in 1999 it appeared in New York [9], rapidly spread across the USA, Mexico, Canada, and the Caribbean [1, 10, 11]. Although the majority of individuals exposed to WNV have asymptomatic or mild infection such as fever and headache, less than 1% of these cases can present with neurological diseases, which includes West Nile poliomyelitis, West Nile encephalitis and West Nile meningitis [12–15]. On the other hand, a lack of an effective prophylactic vaccine or antiviral therapy may lead to more outbreaks of WNV infection [12].

Recently, bibliometric tools have been widely used to investigate the worldwide contributions in many infectious diseases related research including Ebola [16], dengue [17], John Cunningham virus [18], tuberculosis [19], leishmaniasis [20], Zika virus [21], Mayaro virus fever [22], yellow fever disease [23], Malaria [24, 25], toxocariasis [26], campylobacteriosis [27], and Middle East respiratory syndrome coronavirus [28]. Research productivity in WNV field, however, has not been reported to date. The aims of this study are to bibliometrically analyze the quantity and quality of publications indexed in Scopus from different countries to reveal the characteristics of global research output regarding WNV, and to determine the main research topics related to WNV over time.

Findings from this study will provide a holistic picture on WNV-related research which serves as a useful reference for future studies. Furthermore, it gives a picture for authors and editorial journals about future research direction.

## Methods

The database used in this bibliometric study comprises Scopus® (Elsevier BV Company, USA). Scopus is more comprehensive and easier to be used in biomedical field compared to any other tool for literature research, it shows all author ‘s affiliations, and it is considered as the world’s largest database for abstract and citation information [29, 30], that researchers regularly used in various bibliometric studies. “West Nile” and “WNV” were used as phrases to search titles; in addition, “virus”, “fever”, “disease”, “infection”, and “infectious” were used all as words to search titles, abstracts, and keywords; to ensure that the search results adequately reflects the literature related to WNV field. All publications related to WNV were retrieved from the past until the date of December 31, 2016. Data were extracted from Scopus at one day (June 10, 2017) to avoid bias because of daily updating in the database. Data published in 2017 were excluded from the analysis. Search query that is used for data extraction from Scopus database looked like this: ((TITLE (“West Nile”) OR TITLE (*WNV*)) AND (TITLE-ABS-KEY (virus) OR TITLE-ABS-KEY (fever) OR TITLE-ABS-KEY(disease) OR TITLE-ABS-KEY (infection) OR TITLE-ABS-KEY (infectious))) AND (EXCLUDE (PUBYEAR, 2017)).

The methodology applied in this study was comparable to recent bibliometric studies [17, 31–35]. This study was focused on identifying WNV publication trends with regard to publication year, publication type, prolific countries, language of publication, as well as, prolific journals, citations, and collaboration patterns. In this study, two indicators for research evaluation are used, i.e. the impact factor (IF) and the *h*-index. IF is a useful indicator to assess the quality of journals [36, 37]; and *h*-index is used to measure the productivity and impact of published works from different countries [38]. The IF of each individual journal was obtained from the relative official website (i.e. the Journal Citation Reports (JCR 2015) [39]. International collaboration was considered if a publication was co-written by authors from more than one country. If the article was coauthored by researchers from multiple countries, it was calculated for all assigned countries in the article. Additionally, if an article was assigned by author with multiple countries/institutions, it was calculated for all assigned countries in the article. Furthermore, this study determined the contribution of low- and middle-income countries (LMIC) to the WNV literature. The list of LMIC were extracted from the World Bank online databases [40].

## Statistical analysis

Statistical analysis tests were performed using the Statistical Package for the Social Sciences (SPSS) version 15.0. The frequency in count and percentage, summation, and average were used for descriptive statistics. Microsoft Excel 2003 was used for plotting the charts. In addition, to evaluate the growth pattern of research output, linear and exponential regression fitting were compared for the trend in publication. The retrieved publications were divided into three phases according to research trends over time. In this study, frequently used terms were mapped using the VOSviewer software to identify the co-occurrence of title and abstract terms. The co-occurrence networks for the most frequently used terms in the titles and abstracts of the publications related to WNV were studied over the time interval. Each term is demonstrated by a circle, where its diameter and the size of its label illustrate the frequency of the term, and its color reflects most frequently encountered topics in this field [41].

## Results

**Fig. 1:**
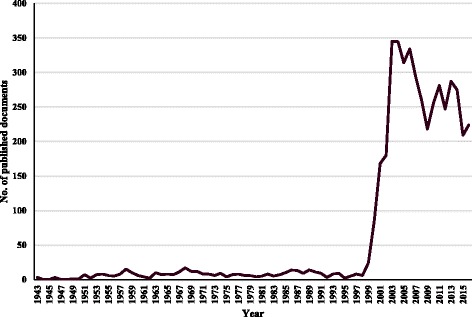
Trends in scientific production, 1943–2016

Thirteen document types were identified in a total of 4729 publications. Articles (3658) were the most commonly used document type which accounted for 77.4% of the total publications. They were followed by reviews (425; 9.0%), short surveys (140; 3.0%), notes (136, 2.9%) and letters (131; 2.8%). For language analysis, 22 languages were used in these publications. English is the dominant language with 4384 (92.7%) publications, followed by French (110; 2.3%), Russian (78; 1.6%) and Spanish (30; 0.6%). The total publications per year are presented in Fig. 1. The results illustrated that the study of WNV reveals an overall increasing trend in the total number of scientific publications from 1943 to 2016. Our results indicate that this trend is best fit by an exponential model (correlation coefficient (r) = 0.775, versus *r* = 0.712 after linear adjustment). A moderate positive significant correlation was found between the years and number of scientific publications (*P* < 0.001). This research trend in the field of WNV can be roughly divided into the following three stages. The first stage was from 1943 to 1999, in which the annual quantity of the number of scientific publications related to WNV published was fluctuated, but was mainly in low rate of research growth. Statistical analysis revealed a low positive significant correlation between the years during 1943 to 1999 and number of scientific publications (*r* = 0.425, *P* = 0.001). The second period was between 2000 and 2006, in which the quantity of publications published was in a rapid increase; with the peak of publications regarding this topic was in 2003 during which the number of scientific publications reached 345 publications. According to the analyzed data there is a moderate positive significant correlation between the years during 2000 to 2006 and number of scientific publications (*r* = 0.873, *P* = 0.01).

In addition, the third stage was from year 2007 to 2016, during which the annual quantity of the number of scientific publications published was fluctuated, indicating a steady-state growth in the field of WNV with a slightly reduction in research rate. Statistical analysis revealed no correlation in the stage between year 2007 and 2016 and number of scientific publications (*r* = −0.394, *P* = 0.265).

The retrieved publications were divided into three periods according to research trends over time, 1943 to 1999, 2000 to 2006, and 2007 to 2016 (Table 1). Among the 4729 publications, 408 (8.6%) were published before 2000 with an annual median growth rate of 7 articles per year [interquartile range: 4–9]; 1771(37.5%) were published from 2000 to 2006 with an annual median growth rate of 314 articles per year [interquartile range: 168–345]; and 2550 (53.9%) publications were published from 2007 to 2016, with an annual median growth rate of 257 articles per year [interquartile range: 222–282].

**Table 1:** Publication quantity and bibliometric indicators stratified by year of publication

Bibliometric indicators	1943–1999	2000–2006	2007–2016
Number of publications	408	1771	2550
Median [IQR] number of publications per year	7 [4–9]	314 [168–345]	257 [222–282]
Correlation coefficient for the changes over time, *P*-value	*r* = 0.425, *P* = 0.001	*r* = 0.873, *P* = 0.01	*r* = −0.394, *P* = 0.265
Total citations	11,193	71,097	41,932
Median [IQR] of citations	9 [1–29]	14 [2–48]	8 [2–20]
*h*-index	52	128	77
Top prolific country	USA with 69 articles	USA with 913 articles	USA with 1322 articles
Top cited article	Lanciotti et al. [50]	Nash et al. [9]	Brass et al. [42]
Top prolific journal	American Journal of Tropical Medicine and Hygiene (19 articles)	Emerging Infectious Diseases (149 articles)	Vector-Borne and Zoonotic Diseases (117 articles)
Top prolific institute	Justus Liebig University Giessen (14 articles)	Centers for Disease Control and Prevention (172 articles)	Centers for Disease Control and Prevention (186 articles)
Number (%) of articles from LMICs	74 (18.1)	91 (5.1)	354 (13.9)

Abbreviations: IQR: interquartile range, LMICs: low- and middle-income countries

Furthermore, a total of 106 countries contributed to the scientific output in the field of WNV research from 1943 to 2016. Contributions from LMIC were considerably small, that is, (*n* = 519; 11%). Table 1 shows the publication quantity and bibliometric indicators stratified by year of publication during 1943–1999, 2000–2006, and 2007–2016.

On the other hand, the publication indicators for the top 10 most prolific countries regarding WNV research are presented in Table 2. The country with the greatest number of scientific publications in WNV research field was the USA with 2304 (48.7%) publications, followed by France with 224 (4.7%) publications, and Canada with 222 (4.7%) publications. The top 10 countries are responsible for 76.2% of the total number of scientific publications. Regarding the international collaboration, the USA collaborated with other countries/territories in 421 publications; which accounted for 18.3% of its total 2304 publications. This was followed by France with 136 (60.7%) publications, and the UK which was collaborated in 87 (64.9%) publications. All publications related to WNV achieved *h*-index of 140 and were cited 124,222 times. The median [interquartile range] number of citations per article thus amounts to 9 [2-28]. The USA had the highest *h*-index of 131, while the other countries into the 10 most prolific countries had an *h*-index between 18 and 40. It is observed that Italy was ranked 4^th^ in regards to the number of scientific publications but only 8th regarding *h*-index; and Israel was ranked 8^th^ regarding the number of scientific publications but 5^th^ in regards to *h*-index.

**Table 2:** The top10 productive countries during 1943–2016

SCR	Country	Number of documents (%)	*h*-index	Collaborations with foreign countries	The number of internationally collaborative publications (%)
1^st^	United States	2304 (48.7)	131	69	421 (18.3)
2^nd^	France	224 (4.7)	40	44	136 (60.7)
3^rd^	Canada	222 (4.7)	35	24	75 (33.8)
4^th^	Italy	161 (3.4)	28	32	68 (42.2)
5^th^	Australia	146 (3.1)	40	19	67 (45.9)
6^th^	United Kingdom	134 (2.8)	32	31	87 (64.9)
7^th^	Germany	121 (2.6)	29	32	68 (56.2)
8^th^	Israel	111 (2.3)	33	11	36 (32.4)
9^th^	Spain	100 (2.1)	26	24	42 (42.0)
10^th^	China	82 (1.7)	18	14	50 (61.0)

Abbreviation: *SCR* Standard competition ranking

More than 26.9% of the WNV related publications were published in the top 10 journals as listed in Table 3. *Emerging Infectious Diseases* is the most productive journal with 227 articles, followed by *Journal of Virology* with 162 articles, *American Journal of Tropical Medicine and Hygiene* with 161 articles, and *Vector-Borne and Zoonotic Diseases* with 157 articles.

**Table 3:** Top 10 most prolific journals (1943–2016) with the total number of papers and IF

SCR	Journal	Frequency (%)	IF^a^
1^st^	*Emerging Infectious Diseases*	227 (4.8)	6.994
2^nd^	*Journal of Virology*	162 (3.4)	4.606
3^rd^	*American Journal of Tropical Medicine and Hygiene*	161 (3.4)	2.453
4^th^	*Vector-Borne and Zoonotic Diseases*	157 (3.3)	1.956
5^th^	*Morbidity and Mortality Weekly Report*	122 (2.6)	10.588
6^th^	*Virology*	120 (2.5)	3.200
7^th^	*Journal of Medical Entomology*	111 (2.3)	1.712
8^th^	*Plos One*	92 (1.9)	3.057
9^th^	*Journal of General Virology*	68 (1.4)	3.192
10^th^	*Journal of The American Mosquito Control Association*	61 (1.3)	0.824

Abbreviation: *SCR* Standard competition ranking, *IF* Impact factor

^a^The impact factor was reported according to the Institute for Scientific Information (ISI) journal citation reports (JCR) 2015

**Fig. 2:**
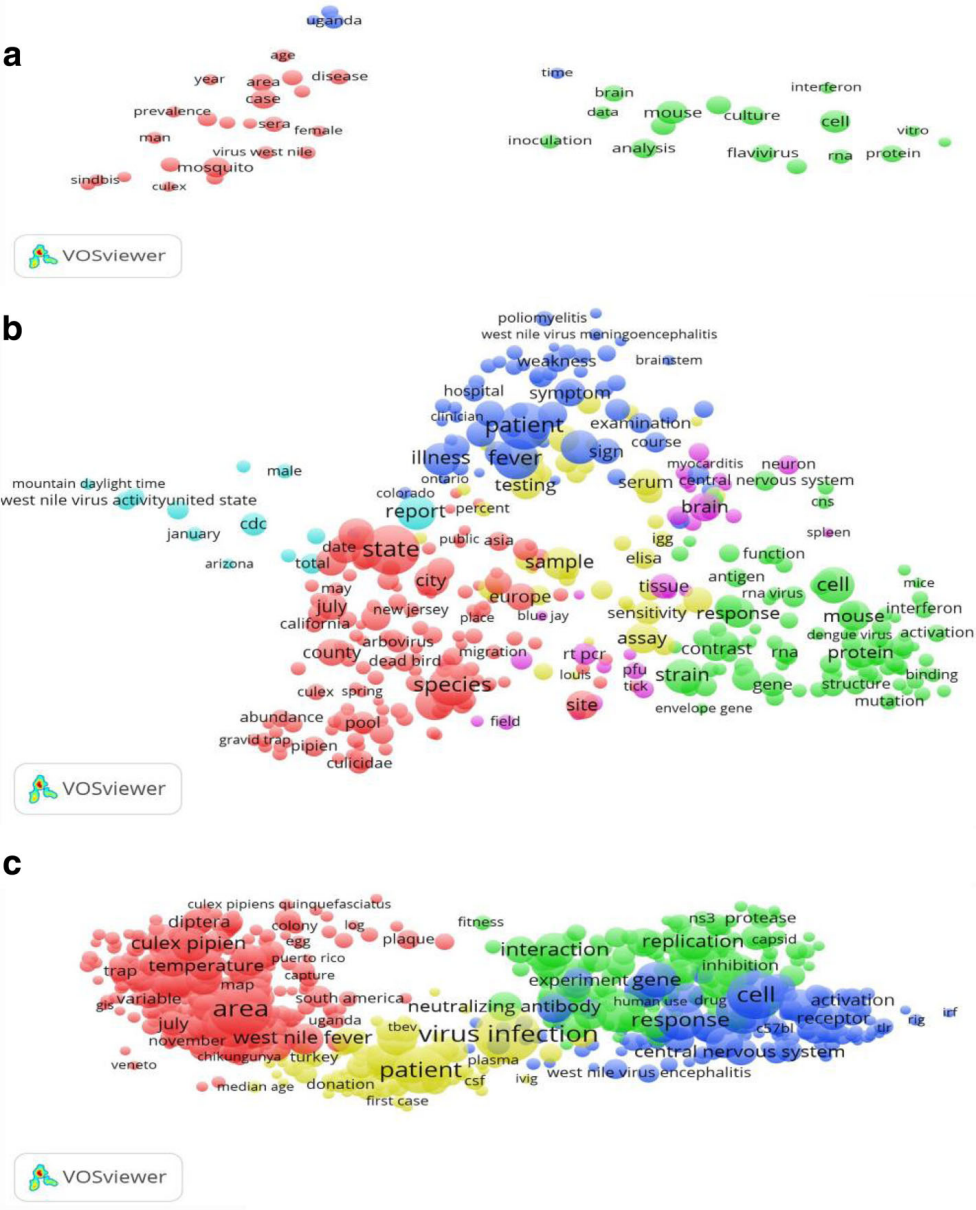
VOSviewer co-occurrence term map. **a** Term map of title and abstract words in West Nile virus publications during 1943–1999 with research topics indicated (based on VOS Viewer). **b** Term map of title and abstract words in West Nile virus publications during 2000–2006 with research topics indicated (based on VOS Viewer). **c** Term map of title and abstract words in West Nile virus publications during 2007–2016 with research topics indicated (based on VOS Viewer)

The maps presented in Fig. 2a, b and c show the most frequently used terms in the titles and abstracts of the publications related to WNV from three specific publication time periods (1943–1999, 2000–2006, and 2007–2016). Figure 2a shows the terms with high co-occurrence frequencies for the period 1943–1999. Here, three loosely connected clusters and scattered were distinguished in which the three most used topics in WNV were signified by three colored clusters: blue, green, and red colors. In this figure, there seems to be two major clusters, one in green color, and one in red color. Green cluster contained terms related to phylogenetic evolution topic, and red cluster contained terms related to transmission topic. Figure 2b shows the terms with high co-occurrence frequencies for the period 2000–2006. Here, six most used topics related to WNV were distinguished which were signified by six colored clusters: blue, green, purple, yellow, cyan, and red colors. In this figure, there seems to be three major clusters, one in green color, one in blue color, and one in red color. Green cluster contained terms related to phylogenetic evolution topic, red cluster contained terms related to transmission topic, and blue cluster contained terms related to signs and symptoms topic. Figure 2c shows the terms with high co-occurrence frequencies for the period 2007–2016. Here four most used topics related to WNV were distinguished which were signified by four colored clusters: blue, green, yellow, and red colors. Blue cluster contained terms related to phylogenetic evolution topic, green cluster contained terms related to transmission topic, yellow cluster contained terms related to signs and symptoms topic, and red cluster contained terms related to ecology and epidemiology of WNV topic.

Table 4 lists the 20 most cited publications [8, 9, 42–59]. The number of citations derived from each work ranges from 1055 to 341. The top 20 publications were published in 12 journals. The *Emerging Infectious Diseases* published the most publications among those top 20 cited publications and also achieved the largest number of citations with 4 publications. This journal was followed by the *New England Journal of Medicine* that generated the largest number of citations with 3 publications with the highest IF value (59.558) among these 12 journals. The most common study design of the list of the most cited publications related to WNV was “laboratory-based research” (11 publications out of 20). On the other hand, Table 5 shows the total publications of the top 10 most prolific institutions. The result designated that *Centers for Disease Control and Prevention* was ranked the first in terms of publication output, followed by *National Center for Emerging and Zoonotic Infectious Diseases*. Among the top 10 institutions, 8 were in the USA, and one each in France and Australia.

**Table 4:** The 20 most frequently cited articles during 1943–2016

SCR	Authors	Title	Year of publication	Source title	Cited by	IF^a^	Study design
1^st^	Lanciotti et al. [50]	Origin of the West Nile virus responsible for an outbreak of encephalitis in the Northeastern United States	1999	*Science*	1055	34.611	Laboratory-based research
2^nd^	Nash et al. [9]	The outbreak of West Nile virus infection in the New York City area in 1999	2001	*New England Journal of Medicine*	789	59.558	A retrospective study
3^rd^	Mackenzie et al. [51]	Emerging flaviviruses: The spread and resurgence of japanese encephalitis, west nile and dengue viruses	2004	*Nature Medicine*	731	30.357	Review
4^th^	Lanciotti et al. [49]	Rapid detection of West Nile virus from human clinical specimens, field-collected mosquitoes, and avian samples by a TaqMan reverse transcriptase-PCR assay	2000	*Journal of Clinical Microbiology*	711	3.631	Laboratory-based research
5^th^	Wang et al. [59]	Toll-like receptor 3 mediates West Nile virus entry into the brain causing lethal encephalitis	2004	*Nature Medicine*	691	30.357	Laboratory-based research
6^th^	Komar et al. [48]	Experimental infection of North American birds with the New York 1999 strain of West Nile virus	2003	*Emerging Infectious Diseases*	683	6.994	Laboratory-based research
7^th^	Hubálek and Halouzka [46]	West Nile fever - A reemerging mosquito-borne viral disease in Europe	1999	*Emerging Infectious Diseases*	667	6.994	Review
8^th^	Campbell et al. [43]	West Nile virus	2002	*Lancet Infectious Diseases*	544	21.372	Review
9^th^	Brass et al. [42]	The IFITM Proteins Mediate Cellular Resistance to Influenza A H1N1 Virus, West Nile Virus, and Dengue Virus	2009	*Cell*	524	28.71	Laboratory-based research
10^th^	Tsai et al. [8]	West Nile encephalitis epidemic in southeastern Romania	1998	*Lancet*	494	44.002	A retrospective study
11^th^	Hayes et al. [45]	Epidemiology and transmission dynamics of West Nile virus disease	2005	*Emerging Infectious Diseases*	466	6.994	Laboratory-based research
12^th^	Iwamoto et al. [47]	Transmission of West Nile virus from an organ donor to four transplant recipients	2003	*New England Journal of Medicine*	459	59.558	Laboratory-based research
13^th^	Petersen and Marfin [54]	West Nile virus: A primer for the clinician	2002	*Annals of Internal Medicine*	438	16.44	Review
14^th^	Pealer et al. [53]	Transmission of West Nile virus through blood transfusion in the United States in 2002	2003	*New England Journal of Medicine*	413	59.558	Laboratory-based research
15^th^	Mostashari et al. [52]	Epidemic West Nile encephalitis, New York, 1999: Results of a household-based seroepidemiological survey	2001	*Lancet*	406	44.002	A cross-sectional study
16^th^	Turell et al. [58]	Vector competence of North American mosquitoes (Diptera: Culicidae) for West Nile virus	2001	*Journal of Medical Entomology*	368	1.712	Laboratory-based research
17^th^	Turell et al. [57]	An update on the potential of North American mosquitoes (Diptera: Culicidae) to transmit West Nile virus	2005	*Journal of Medical Entomology*	354	1.712	Laboratory-based research
18^th^	Rappole et al. [55]	Migratory birds and spread of West Nile virus in the Western Hemisphere	2000	*Emerging Infectious Diseases*	352	6.994	Review
19^th^	Sejvar et al. [56]	Neurologic Manifestations and Outcome of West Nile Virus Infection	2003	*Journal of the American Medical Association*	347	37.684	A prospective study
20^th^	Diamond et al. [44]	B cells and antibody play critical roles in the immediate defense of disseminated infection by West Nile encephalitis virus	2003	*Journal of Virology*	341	4.606	Laboratory-based research

Abbreviation: *SCR* Standard Competition Ranking

^a^The impact factor was reported according to the Institute for Scientific Information (ISI) journal citation reports (JCR) 2015

**Table 5:** Top 10 most productive institutes (1943–2016)

SCR^a^	Institute	Country	*n* (%)
1^st^	*Centers for Disease Control and Prevention*	USA	362 (7.7)
2^nd^	*National Center for Emerging and Zoonotic Infectious Diseases*	USA	232 (4.9)
3^rd^	*New York State Department of Health*	USA	170 (3.6)
4^th^	*UT Medical Branch at Galveston*	USA	160 (3.4)
5^th^	*Colorado State University*	USA	134 (2.8)
6^th^	*Washington University in St. Louis*	USA	130 (2.7)
7^th^	*Wadsworth Center for Laboratories and Research*	USA	129 (2.7)
8^th^	*UC Davis*	USA	118 (2.5)
9^th^	*Institut Pasteur, Paris*	France	84 (1.8)
10^th^	*University of Queensland*	Australia	78 (1.6)

## Discussion

This study sheds light on how the status of research regarding WNV has developed during the past 73 years. The study also recognized the main research topics related to WNV over time. The bibliometric analysis of this study shows that the number of scientific publications concerning WNV increased in the last 73 years. These results on WNV are consistent with those obtained from similar previous analysis of the literature related to other infectious diseases [16–24]. This progress may be attributed to the increase in the awareness of the importance of research in the field of WNV [60–64]. The annual quantity of the number of scientific publications related to WNV published has fluctuated (up and down) in the last 15 years. The annual quantity of literature published before 2000 followed a low rate of research growth; while the quantity of publications after 2000 were published in a stage of rapid development. For instance, the steeper increase in the 2000s reflects the spread of virus during those years, when the WNV disease was limited to Mediterranean region, Africa, Asia, and some part of east Europe [6–8] prior to the end-1990s. In addition, WNV was believed to have a minor risk for human, until an outbreak in New York city in 1999 [9], and over the next year, WNV quickly spread across the continental USA, Latin America, Canada, and the Caribbean islands [1, 10, 11]. In contrast, the higher publication rate in the 2000s could be related to the interest for new diagnostic procedures [49], new therapeutic strategies [65], and effective preventive measures [66].

During this 73-year period, the USA had ranked at the top regarding the quality and quantity of published publications. Although it should come as no surprise that the USA lead the world in research production in WNV, which has been found in numerous previous biomedical studies [17, 31, 33–35, 67–69], it is surprising that relatively small countries compared to the USA in their population size such as France, Italy, Australia, Germany, Israel, and Spain lead the world in research production in WNV. In seeking explanation for these findings, the most possible one may be related to high prevalence of cases with WNV in these countries which experienced several outbreaks [45, 70, 71]. Another possible clarification for these results could be attributed to spending on research and development, gross domestic product per capita, indexed scientific journals, number of universities, and a good utilization of resources for these countries assigned to research output [72–74]. Another important finding was that the USA also has the highest *h*-index, which demonstrated that WNV publications originating from the USA had the highest quality.

To the best of knowledge, this study is the first of its kind that assessed research productivity in the field of WNV during the period between 1943 and 2016 at global level. However, there were some limitations for this study and most of them were similar to previous bibliometric studies [17, 31, 33–35, 68, 69]. This study was limited because only publications that were contained and ranked within Scopus were analyzed. Furthermore, data in 2016 may be incomplete, because some of the latest data from 2016 may not have been uploaded to the database at the day of data extraction.

## Conclusions

Although bibliometric analyses have been performed across several fields of infectious, this study may be believed to be the first study regarding WNV. In conclusion, bibliometric analyses were performed to provide estimates of research productivity related to WNV at global level. There is an obvious trend of WNV research after 2000, and countries with high income tend to have more contributions in the field of the WNV research. The research output is low among LMIC. The USA, France, Canada, Italy, Australia, the UK, Germany, Israel, Spain, and China had high productivity in publications related to WNV. The USA produced the largest number of scientific publications. The *US Centers for Disease Control and Prevention* obtained the leading position among the institutions in terms of publication output. Furthermore, “phylogenetic evolution”, “transmission”, “signs and symptoms” and “ecology and epidemiology” may be the latest hot spots in the field of WNV, and related researches may be pioneers to direct this field in the next few years. In general, this study not only presents a full view of global WNV research, but also can contribute for future further research in this field.

## Abbreviations

IFs: Impact factors
IQR: Interquartile range
ISI: Institute for Scientific Information
JCR: Journal Citation Report
LMICs: Low- and middle-income countries
SCR: Standard competition ranking
SPSS: Statistical Package for Social Sciences
WNV: West Nile virus
